# Founder Effects in Hereditary Hemorrhagic Telangiectasia

**DOI:** 10.3390/jcm10081682

**Published:** 2021-04-14

**Authors:** Tamás Major, Réka Gindele, Gábor Balogh, Péter Bárdossy, Zsuzsanna Bereczky

**Affiliations:** 1 Division of Otorhinolaryngology and Head & Neck Surgery, Kenézy Gyula Campus, University of Debrecen Medical Center, H-4031 Debrecen, Hungary; 2 Division of Clinical Laboratory Science, Department of Laboratory Medicine, Faculty of Medicine, University of Debrecen, H-4032 Debrecen, Hungary; gindele.reka@med.unideb.hu (R.G.); balogh.gabor@med.unideb.hu (G.B.); 3 Hungarian Heraldry and Genealogical Society, H-1014 Budapest, Hungary; peter@bardossy.hu

**Keywords:** hereditary hemorrhagic telangiectasia, germline mutation, founder effect, haplotype, genealogy, population genetics

## Abstract

A founder effect can result from the establishment of a new population by individuals from a larger population or bottleneck events. Certain alleles may be found at much higher frequencies because of genetic drift immediately after the founder event. We provide a systematic literature review of the sporadically reported founder effects in hereditary hemorrhagic telangiectasia (HHT). All publications from the *ACVRL1*, *ENG* and *SMAD4* Mutation Databases and publications searched for terms “hereditary hemorrhagic telangiectasia” and “founder” in PubMed and Scopus, respectively, were extracted. Following duplicate removal, 141 publications were searched for the terms “founder” and “founding” and the etymon “ancest”. Finally, 67 publications between 1992 and 2020 were reviewed. Founder effects were graded upon shared area of ancestry/residence, shared core haplotypes, genealogy and prevalence. Twenty-six *ACVRL1* and 12 *ENG* variants with a potential founder effect were identified. The bigger the cluster of families with a founder mutation, the more remarkable is its influence to the populational *ACVRL1/ENG* ratio, affecting HHT phenotype. Being aware of founder effects might simplify the diagnosis of HHT by establishing local genetic algorithms. Families sharing a common core haplotype might serve as a basis to study potential second-hits in the etiology of HHT.

## 1. Introduction

### 1.1. Definition

A founder effect may result from the establishment of a new population by individuals deriving from a much larger population (a true founder event) or an extreme reduction in population size (a bottleneck event). As a consequence, certain alleles may be found at a higher frequency than previously and can reach even a higher prevalence by genetic drift in the period immediately after the founder event, and later, by inbreeding, particularly in population isolates [1].

### 1.2. Founder Effects in Population Isolates

Population isolates serve as an excellent basis for the investigation of founder effects. They exist in isolation from other populations as a result of cultural (linguistic or religious) or geographical (mountains, seas, deserts, etc.) barriers [2]. The best-known cultural population isolates are the Ashkenazi Jews and North American Anabaptist groups (the Mennonites, the Hutterites and the Old Order Amish). Each of these is characterized by little genetic inflow, identifiable small founding population and well-known historical bottleneck events, high standard of living, high interest in illness and highly accessible medical care [3,4]. The Anabaptist communities, furthermore, keep extensive genealogical records, live in large families with low rates of non-paternity and high rates of consanguinity and have notably uniform socioeconomic circumstances [3,5].

As a result of founder effects, both the cultural and geographical population isolates have their characteristic Mendelian (autosomal recessive, autosomal dominant and X-linked) or mitochondrial disorders. The increased incidence of these otherwise rare conditions allows for linkage analysis and identification of causative genes [3,6]. Founder alleles might contribute to the risk for more common complex diseases, like type II diabetes, obesity or bipolar affective illness in the Mennonites and Amish [5,7]. Moreover, several population isolates exhibit peculiar founder germline dominant *BRCA1/2* alleles with early onset breast and ovarian cancer risks [4,8,9]. Consequently, population isolate-specific databases and screening panels for genetic disorders might be established [4,10].

### 1.3. Pioneer Reports for Founder Effects in Hereditary Hemorrhagic Telangiectasia

The majority of familial (germline) vascular malformations or syndromes are inherited in an autosomal dominant trait and mutations are usually family specific [11]. Although, per definitionem, it is considered to be a rare disease (with a prevalence beneath 1 in 2000) [12], hereditary hemorrhagic telangiectasia (HHT) is the most common inherited arteriovenous malformation syndrome [11]. The so-far identified causative genes are *ENG* and *ACVRL1* (accounting for HHT1 and HHT2, respectively, over 85% of all HHT cases), *SMAD4* (JP/HHT phenotype, 2% of HHT cases) and *GDF2* (HHT5, reported occasionally) [13]. The worldwide prevalence of HHT is 1:5000–1:10,000 [14]. However, this widely accepted value is an estimate. Prior to the identification of *ENG* and *ACVRL1* in the mid-nineties [15,16], direct questionnaire (addressed to general practitioners and specialists) and/or hospital record-based methods were performed to assess the prevalence of HHT, with variable return rates and results (2.5–19.4 per 100,000) [17,18,19,20,21]. Each author highlighted that these results were underestimates. Despite the variable prevalence rates, population genetic studies reported some geographical regions with prominently high point-prevalences, like Ain, Jura and Deux-Sevres Counties of France or the islands of Curacao and Bonaire of the Netherlands Antilles [18,22]. Subsequent comprehensive molecular genetic studies showed unrelated families within these areas with identical *ENG* and *ACVRL1* mutations and shared adjacent core haplotypes, suggesting common ancestry [23,24,25]. If one or few of these variants with common ancestry dominate a geographic area, its founder effect is confirmed.

In the present study, we provide a systematic literature review of founder effects in HHT, reported in the past two decades.

## 2. Study Design

### 2.1. Literature Search

**Figure 1:**
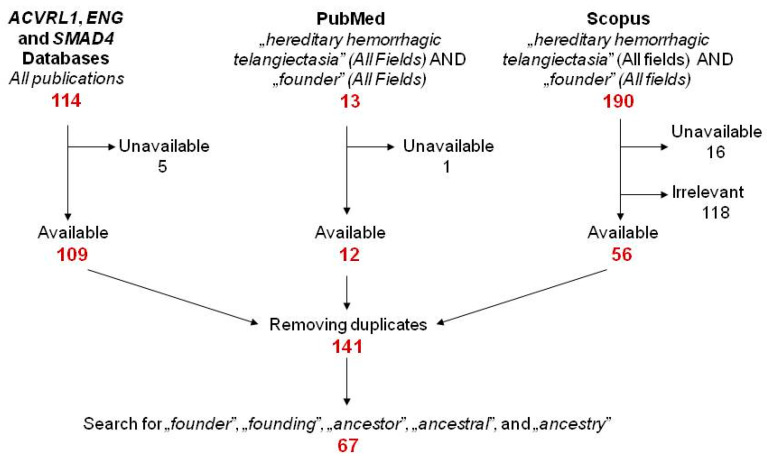
The algorithm of the literature search for hereditary hemorrhagic telangiectasia (HHT) founder effects.

The targets of the literature search were (1) all publications referred in the *ENG*, *ACVRL1* and *SMAD4* Databases, respectively [26,27,28]; (2) results from PubMed and Scopus for *“hereditary hemorrhagic telangiectasia” (all fields)* AND *“founder” (all fields)* (both databases were accessed on 9 February 2021). Following the removal of papers considered as irrelevant based on their abstracts and duplicates from the primary pool, the whole text of 141 publications was subsequently searched for the terms *“founder”* and *“founding”* and the etymon *“ancest”*. The resulting 67 papers (listed in Supplementary Materials Table S1) and if required, their references were independently searched for HHT founder mutations by the authors T.M. and R.G. (Figure 1).

### 2.2. An Arbitrary Grading System to Assess Evidences for Founder Effect in HHT

In the aforementioned areas with elevated HHT point-prevalence, geographical barriers (island or mountains) are suspected. However, in the midst of expanding transport potentials in the 20th and the 21st century the significance of geographical barriers as the main reason for population isolates is declining. On the other hand, HHT is not detected in known cultural population isolates. Therefore, we assume that the magnitude of a founder effect in HHT is also continuously declining even in previous geographical population isolates, essentially by emigration and immigration [25]. By reviewing the literature, pieces of evidence for founder effects were collected as follows: (1) if identical causative mutations are detected in unrelated families by a laboratory with expertise in HHT genetics; (2) if families with an identical mutation originate from or live in the same geographical area; (3) if there is genealogical evidence of common ancestry; (4) if shared core haplotypes are detected by intragenic and flanking extragenic polymorphic markers; and (5) if the investigated mutation is still prevalent in the given geographical area, thus responsible for the majority of causative variants. The simultaneous reports of shared core haplotype and shared area of ancestry/residence as pieces of evidence for a founder effect were especially frequent.

Somewhat arbitrarily, we constructed a grading system to assess founder effects in HHT (Table 1). Criteria were weighted by the number of kindreds reported. The terms “apparently unrelated families”, “shared area of ancestry or residence”, “shared core haplotype” and “genealogical evidence of common ancestry” were not uniformly and unequivocally defined in the majority of papers. In these cases, we accepted the authors’ self-report. Although a core haplotype shared by a few (2–4) families only, refers to their potential common ancestor, from a population genetic point of view; however, it is not strong enough to prove the founder effect. If these families still live in or originate from the same area, it might be a stronger argument at the founder effect. Unrelated families were defined only in two papers, as “not related by blood within living memory” [22], or “unrelated going back for at least four generations” [29]. Here we define a mutation as “locally still prevalent”, if it accounts for ≥30% of all HHT families or cases in an administrative area (minimum a district of a county) or distorts national HHT mutation distribution by presenting in a ≥10% proportion.

If a variant fulfilled the criteria of a minimum grade II founder effect in any of the publications, it was collected, even the absence of authors’ self-report as a founder. Furthermore, all other available reports of the captured minimum grade II founders were reviewed.

At the assessment of mutation pathogenicity, we accepted authors’ self-report. In the case of variants indicated as “pending” in the *ENG* and *ACVRL1* Databases [26,27], we reassessed variant pathogenicity by in silico prediction analyses and authors’ arguments (co-segregation, absence in healthy controls, comparison with ortologs, etc.) (Supplementary Materials Table S2). At variant classification, we followed the joint consensus recommendations of the American College of Medical Genetics and Genomics (ACMG) [30].

## 3. The Overview of Potential Founders

### 3.1. Variant Distribution, Type and Pathogenicity

A total of 26 *ACVRL1* and *12* ENG variants (Table 2) were identified with grade I to IV founder effects. The *ACVRL1* c.1445C>T variant was assessed as benign and excluded from further analysis.

Neither the *SMAD4* variants associated with the HHT or JP/HHT phenotype nor the extremely rare *GDF2* were reported as founders [72,73].

**Figure 2:**
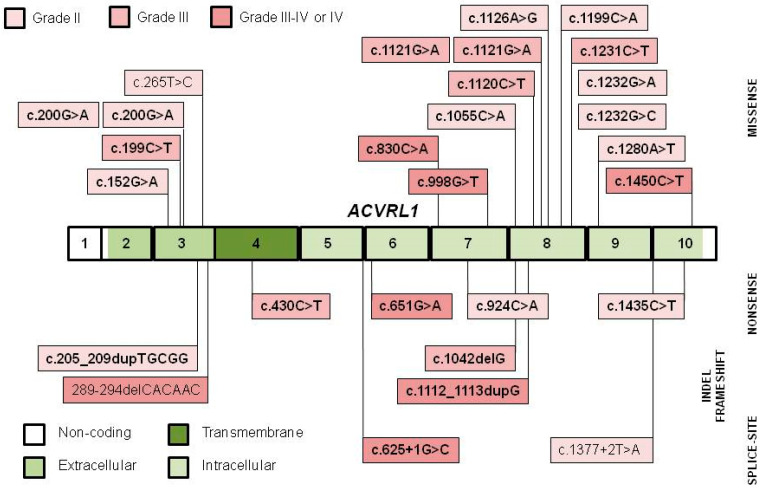
The grade II to IV founder variants in the exons and flanking intronic regions of *ACVRL1*. Pathogenic variants are indicated in bold. In the case of the c.200G>A and the c.1121G>A variants, two distinct flanking haplotypes were identified [25].

The distribution of founders throughout the *ACVRL1* and the *ENG* genes is similar to all mutations available in the databases [26,27]. The majority (16/26) of *ACVRL1* founder variants (Figure 2) is missense type, clustering within exons 3 and 8. Missense variants in exon 3 of the extracellular domain might impair TGF-β receptor type I and II interactions and ligand-dependent signaling [43]. Missense variants in exon 8 involve highly conserved amino acids (c.1120C>T and c.1121G>A affect Arg in codon 374, while c.1231C>T, c.1232G>C and c.1232G>A affect Arg in codon 411) within the core of the intracellular kinase C-lobe, compared to ortologs and paralogs [24,39,43,48,52,54]. Codons 374 and 411 are considered as mutation hot-spots, but several of them appear as grade II or even grade III founders in distinct geographical areas (Table 2) [24,25]. Interestingly, all but three of the variants (88.5%) were pathogenic, substantially exceeding the 64.5% given in the *ACVRL1* Database [27].

**Figure 3:**
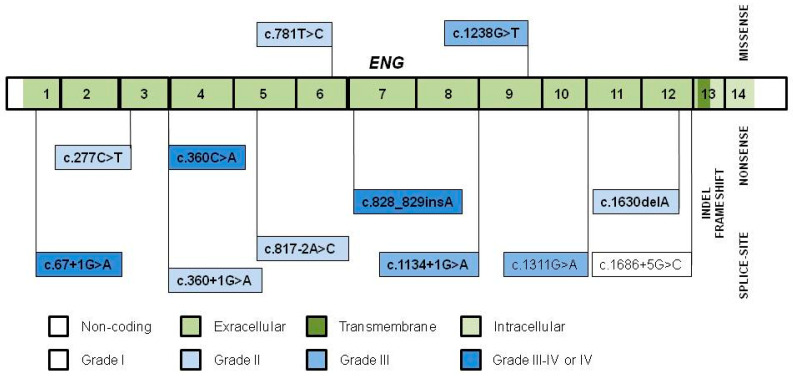
The grade II to IV founder variants in the exons and flanking intronic regions of *ENG*. Pathogenic variants are indicated in bold.

Founder *ENG* variants (Figure 3) tend to localize throughout its exons encoding the extracellular domain, with more nonsense, frameshift and splice-site and less missense variants [24,43,48,54,67]. Ten out of the 12 variants (83.3%) are pathogenic, similarly to the 78.9% given in the *ENG* Database [26].

Although founder effects and hot-spots are not excluding terms, we suppose that max. grade II variants with several independent reports (the *AVCRL1* c.152G>A, c.200G>A, c.924C>A, c.1232G>A and c.1435C>T; and the *ENG* c.277C>T and c.360+1G>A in Table 2), are rather hot-spots than founders and accordingly, were not considered as founder variants by the authors, either. Grade III or IV variants with several independent reports (the *ACVRL1* c.199C>T, c.430C>T, c.1120C>T, c.1121G>A, c.1231C>T and c.1450C>T in Table 2) might be hot-spots with local founder effects, in agreement with the authors.

### 3.2. Grade IV Founder Variants

Henceforth, we focus on the *ACVRL1* and *ENG* variants with unequivocal founder effects.

We detected an identical *ACVRL1* c.625+1G>C pathogenic splice-site mutation in 19 individuals of five unrelated families in Heves County, Hungary [46]. Neither the probands nor their available alive kinships were aquainted with the others. Haplotype analysis with a total of eight intragenic and flanking extragenic polymorphic markers showed correspondent haplotypes at the tested region of the mutant chromosome. Subsequent genealogical analysis revealed a marriage from 1779 as the potential common ancestor of families. According to our stratified population screening study performed in order to assess HHT point-prevalence in the primary attendance area of the Ferenc Markhot County Hospital, Eger, Hungary (population of 225,000 in May 2017), this mutation dominated the study area by 57.7% (15/26 HHT patients) [47]. Currently, 21 tested heterozygous individuals of seven families constitute this kindred, still with a correspondent core haplotype (Supplementary Materials Figure S1). A non-complete geographical isolation given by the underdeveloped road network and the very low standard of living up to the beginning of the 20th century is considerable.

The *ACVRL1* c.651G>A pathogenic nonsense variant was detected in 26 patients of seven families from Østfold (Southeastern Norway) and neighboring West Sweden. Haplotype analysis was performed in five of these families, showing a common core haplotype [48]. From a nationwide point of view, this mutation accounts for 6.2% (6/113) of all and 7.2% (7/97) mutation positive HHT families and 11.1% (26/234) of HHT patients in Norway.

The *ACVRL1* c.830C>A (p.Thr277Lys) pathogenic missense variant was detected in 24 Norwegian families, with 22 of them originating from Rana District in Nordland County. Haplotype analysis was performed in 13/24 families, showing a shared core haplotype. This mutation dominates HHT in this geographical area, and furthermore, accounts for 21.2% (24/113) of all HHT families, and additionally, 24.7% (24/97) of HHT families and 24.8% (58/234) of HHT patients with an identified pathogenic or likely pathogenic mutation in Norway [48]. The area’s localization in the proximity of the Arctic Circle and its landscape consisted of fjords and mountains might have served as potential causes of a past geographical isolation.

The *ACVRL1* c.1112_1113dupG pathogenic frameshift variant was initially described in 2003, by Abdalla et al., in 8 of 15 individuals of a family originating from the Rhône-Alpes region, France [43,51]. One year later, Lesca et al. reported this variant in 17 unrelated index cases [24], collected through the French HHT network. In microsatellite studies all patients shared a common haplotype. At that time, this variant accounted for 17% (17/100) of all identified family-specific HHT mutations, appreciably skewing the nationwide *ACVRL1/ENG* ratio. By 2008, already 35 families were known with this variant, still with a common core haplotype and an estimated age of the most recent common ancestor of 325 years. Although not exclusive in the area, this mutation is dominating HHT in Ain and Jura of the Rhône-Alpes region, with its epicenter to the Valserine valley [22,25]. The authors speculate that the founder event might have occurred in this region, prior to the 17th, century either as a de novo variant or by immigration, and subsequently, it increased in prevalence due to genetic drift in the non-perfect geographical population isolate of the Valserine valley [25], with a contribution of the relatively high level of geographical endogamy [74]. Finally, this variant spread within and outside the Rhône-Alpes region [25]. The mutation was also reported from clinical centers of Europe and North America (it might be identical with the original French cluster) [51], and also from Utah, US, in a family with ancestry to Ain, France [54]. Up to the present day, all families with c.1112_1113dupG variant can be traced back to the Rhône-Alpes region, confirming its founder effect.

The *ENG* c.67+1G>A pathogenic splice-site variant was detected in 7/10 unrelated families in the Curacao and Bonaire islands (population of 116,000 and 11,800 in 1998, respectively) of the Netherlands Antilles. The seven kindreds consisted of 58 affected individuals and 47 participated in the study of Gallione et al. [23]. Each proband had a shared core haplotype. At the time of the study, 102 HHT patients from 23 kindreds were living in Curacao and Bonaire, corresponding to a very high point-prevalence of 1:1331. In addition, this was an obvious underestimate, as only individuals above 12 years were assessed and only 70% of all known family members participated in the stratified population screening in the Afro-Caribbean population of the Netherlands Antilles [75]. The Dutch gained control over the temporarily nearly uninhabited islands in the middle of the 16th century and Curacao soon became the center of the Caribbean slave trade. The mutation could have been either of Antillean or African origin, although HHT is very rare in Sub-Saharan Africa [75,76]. The island as a past geographical population isolate and the relatively young age of the population are obvious. The variant was later also detected in the Netherlands in a family of Antillean origin [52].

The *ENG* c.360C>A pathogenic nonsense variant with a shared core haplotype was initially detected in 7/14 probands (50%), and furthermore, in 36/56 HHT patients (64.3%) with identified mutations from the island of Funen (Fyn), Denmark with a population of 470,000 in 1999 [44,66]. Mutation is estimated to have occurred 13 or 14 generations (approximately 340 years) before, either as a new variant or by immigration. Ten years later, in a Danish national HHT mutation study, 13 unrelated families were reported with this variant, that therefore, accounts for 13.68% (13/95) of all Danish kindreds with identified mutations. Otherwise, HHT point-prevalence was not extremely high in Funen (1:6410), as assessed by proband recruitment from hospital discharge records and subsequent family screening [21].

### 3.3. Potentially Grade IV Founder Variants

Five families and 38 affected individuals with the *ACVRL1* c.998G>T pathogenic missense variant were reported from Utah, US [50]. Genealogy revealed a common ancestor born in the early 1800s and his over 3000 at-risk living descendants. The local prevalence is unknown. This variant was detected in an additional American HHT family with four clinically affected individuals [43,51]. This family is geographically linked to the large Utah kindred, suggesting their common ancestry. A past geographical (varied landscape) or a cultural (relative young population by Mormon settlements in the mid-19th century) isolation might have occurred in Utah, but these are not referred by the authors.

Five families with 20 patients from two locations of Nordland, Norway, share the *ACVRL1* c.1450C>T pathogenic missense variant [48]. We have no information about the HHT prevalence in Nordland.

The *ENG* c.289-294delCACAAC indel variant was detected in 10 families with 40 affected individuals, residing in Bergamo County, Italy [29]. The authors emphasize that other HHT families with different mutations are also found in the population of 1,021,700 in 2007. Although very presumable, we cannot classify it straightaway as a grade IV variant in the lack local HHT prevalence data at that time. A past geographical isolation offered by the Alps in the northern part of the area is likely.

The *ENG* c.828_829insA variant was detected in six individuals of two large families with probands living in County A (population of 170,000 in 2002), Akita Prefecture, Japan [70]. A total of 15 patients in the two families were alive at the completion of the study. On the other hand, 23 known HHT patients were living in County A at that time, giving an approximate point-prevalence of 1:8000. We have no information about the number patients from the two concerned kindreds living in the study area. The study population is otherwise a past geographical isolate with founder effects of various genetic diseases like cholesteryl ester transfer protein (CETP) deficiency [77].

### 3.4. Tracing the Founder Event

Besides the above *ACVRL1* c.998G>T and c.1112_1113dupG variants, identical mutations with shared core haplotypes are sporadically observed in distinct populations. One of the two haplotypes of the *ACVRL1* c.200G>A (p.Arg67Gln) and one of the three haplotypes of the c.1120C>T (p.Arg374Trp) variants and the c.430C>T (p.Arg144*) variant are shared by French and Italian patients, and in the case of the latter two, the *DS12S1677* and *D12S296* marker alleles in the partially shared core haplotype are absent in the French control population. Thus, the common origin of these variants by Italian immigration to France, is likely [25].

The *ACVRL1* c.1121G>A (p.Arg374Gln) was initially described by Abdalla et al. [43], and later the variant was reported in three unrelated families in the US and France each, with ancestry from Deux-Sevres, France, in both cases. The French authors detected a common core haplotype. Based on the shared authorship of these publications, it is conceivable that these families are identical [25,51].

The *ACVRL1* c.1232G>A (p.Arg411Gln) variant is detected in two distantly related North American families with German and French origins, respectively [51]. Indeed, this variant was later reported from France and Germany [24,25,41]. On the other hand, it is present in several other unrelated populations, confirming a mutation hot-spot [38,39,60,62].

Two Antillean kindreds (four affected individuals) and a Dutch kindred (seven affected individuals) shared the *ENG* c.1238G>T missense mutation with a common core haplotype [23,78]. The Dutch family originates from Zeeland, the westernmost province of the Netherlands with extensive trading with the West Indies in the colonial times. The authors argue a potential European origin of this Antillean HHT mutation. The variant is reported from the Netherlands in another study of Letteboer et al. [52]. The European origin is further confirmed by the aforementioned fact that HHT is extremely rare in Sub-Saharan Africa [76].

### 3.5. Mutation Age

In order to estimate the age of the different founder events, likelihood-based methods were developed. Estimation is based on the size of the shared core haplotype flanking the mutation in the affected individuals, considering allele frequencies of the markers in the study population, likely recombination positions and mutation rates [25,44]. The mutation age is given with a confidence interval; the latter is highly influenced by the number of probands [25]. Two of the five founder variants with age estimates in the study of Lesca et al. showed regional clustering. One haplotype of the *ACVRL1* c.1121G>A (4 generations) in Deux-Sevres County was the youngest of all, while in the case of the c.1112_1113dupG (13 generations) variant in Jura and Ain Counties, the past partial geographical isolation could account for the still-existent clustering [25]. This might explain the dominance of the *ENG* c.360C>A variant with the same age in the island of Funen, Denmark, too [44]. In the case of the remaining three founder variants in the French study with similar age or even older [25], one possible reason for the missing regional clustering might be the lack of past population isolation.

### 3.6. The Contribution of Genealogy

The main sources of genealogical tree reconstruction are parish registers (birth/christening, marriage and burial/death) and civil records from the late 17th century [74]. By means of genealogy, Letteboer at al found the common ancestor in three of the eight families with the *ENG* c.781T>C variant in 1765, in five of the seven families with the *ACVRL1* c.1042delG variant in 1722 and in all five families with the *ENG* c.1311G>A variant in 1745, respectively [52]. The six families with the *ACVRL1* c.625+1G>C variant and its shared core haplotype could be traced back to a single founder in 1779 [46]. A co-existence of several favorable circumstances supported our effort. First, original parish registers were not destroyed by wars or natural forces. Second, in Hungary surnames are identical with family names (and not patronymics, for instance). Our HHT kindreds beared infrequent family names. Third, non-paternity probably did not occur in our series. Fourth, pedigree charts of the six families met at a time following the introduction of parish registers. Though prescribed by Pope Pius IV in the Council of Trent in 1563 [79], Catholic church registers were introduced at the beginning of the 18th century in Hungary. We emphasize that the identified common ancestor of the concerned families is not inevitably identical with the founder of the mutation in the population. Further families with this variant sharing a less correspondent haplotype might be identified whenever in the future, suggesting a more remote common ancestor [46].

However, many genealogical links might be found between affected families, resulting in a non-absolute genealogical convergence of the ancestors [22,74]. Tracing the pedigrees of 49 families living or originating in five contiguous villages of the Valserine Valley, 47,000 parish and 6000 civil records covering three centuries were reviewed, resulting in a dense network of common ancestors. Finally, 929 individuals in the mid-17th century were found at the top of the genealogical trees. The possible founder(s) should be among them [22]. The genetic tests performed more than a decade later revealed several causative variants in this area, in addition to the French founder one. An additional example for the multiple common ancestries of two Hungarian HHT families with the *ENG* c.817-2A>C mutation is shown in Supplementary Materials Figure S2 [42]. In conclusion, in the molecular genetic era, genealogy plays an auxiliary role in the identification of founder effects. We propose the thorough medical pedigree construction up to the farthest ancestors with HHT symptoms by alive kindred individuals’ hearsay and keeping of registers with HHT family names.

### 3.7. How Can an Autosomal Dominant Disease Like HHT Result in a Founder Effect?

The fate of a potential founder allele is a combination of genetic drift and selection. There is evidence for some of the frequent founder mutations in recessive diseases to confer a selective advantage to heterozygotes (e.g., the G6PD^Med^ mutation resulting in malaria resistance) [4]. Selection might have a neutral or a slight negative impact on the founder effects in HHT. In a population genetic study performed by Kjeldsen et al. in Funen, Denmark, more than two decades ago, HHT was found to associate with an excess mortality, particularly in patients below the age of 60 years at inclusion. In this latter group, the cumulative mortality rate was twofold of the age- and gender-matched controls, and this rise was exclusively attributable to HHT complications as bleeding and pulmonary arteriovenous malformations (PAVMs) [21]. Major complications from undiagnosed PAVMs like paradoxical emboli (leading to ischemic stroke or brain abscess) or hemothorax significantly reduce the quality of life and life expectancy of HHT patients [80,81]. On the other hand, penetrance is age-related, and these complications have no significant effect on reproductive fitness, permitting HHT to result in a founder effect. This might be particularly the case in HHT2 with occasionally later onset of symptoms [46,50,82] and definitely lower prevalence of potentially fatal PAVMs, compared to HHT1 [45,54,67]. Indeed, two-thirds of the founder mutations affect the *ACVRL1* gene.

## 4. The Significance of Founder Effects

The bigger the cluster of HHT families and patients with a founder variant, the more remarkable is its influence to the HHT1/HHT2 ratio in a certain administrative area [23,24,46,47,48,66] or even nationwide, slightly affecting the HHT phenotype [29,48,67]. On the other hand, the phenotypes of HHT1 and HHT2 are overlapping, and according to the International HHT Guidelines released in 2011 and its 2020 revision [83,84], neither the diagnostic nor the management algorithms differ in HHT1 and HHT2.

Similar to the second-hit hypothesis of familiar cancers, an environmental (inflammation, hypoxia, sunlight, trauma) or a genetic (a somatic mutation in the wild-type allele of HHT genes or a germline variant in modifiers) second-hit added to the germline HHT mutation [85], might explain the focal appearance of vascular lesions, the age-related penetrance and the considerable intrafamilial variance in HHT phenotype [23,50,52,66]. Founding kindreds, especially the larger ones with more uniform core haplotypes and living in uniform environmental conditions, might be theoretical targets for these genetic second-hit studies.

When one or a few founder variants account for the majority of all pathogenic variants found in a population, testing for the founder(s) may be performed first [10]. Considering the International HHT Guidelines [84], this screening might refer exclusively to the pathogenic variants and each of them appearing in the study area must be tested simultaneously. However, relying on merely to the founder screening carries some risks. Non-founder variants also presenting in the area [25,66,70] might be overlooked. Furthermore, in the nationwide study of Heimdal et al. [48], a family with the pathogenic missense *ACVRL* c.1450C>T founder and a likely pathogenic *ACVRL1* c.11delG variant in cis were reported. This might be also overlooked by screening for founder mutations only. With the availability of Next-Generation Sequencing, many labs would find it cost and time efficient to sequence the entire gene(s). In our practice, we screen for the known local founder mutations in new families with HHT phenotype as first-line test and accept them as the causative variants in the case of co-segregation. If no founder mutation is found in the patient, second-line genetic investigations including all HHT causative genes are performed.

## 5. Limitations

The references in the HHT Mutation Database are not necessarily up-to-date, as a number of recently detected HHT variants are not submitted. A part of these variants could have been missed by the subsequent PubMed and Scopus search, especially the ones that were not considered as founder by the authors.

With very few exceptions, all the listed data (Table 2) originate from European or North American subpopulations, resulting in a publication bias.

The ambiguous definitions of “apparently unrelated families”, “shared area of ancestry or residence”, “shared core haplotype”, “genealogical evidence of common ancestry” and the arbitrary grading system could either under- or overestimate certain variants as founders. The definition of the “founder effect” itself and, furthermore, the minimal population size for the investigation of founder effects are equivocal in the literature. Theoretically a single large resident family with an exactly kept genealogical tree might result in a founder effect in a small population, especially if it is thoroughly screened, like the kindred with the *ACVRL1* c.1120C>T in the paper of Kjeldsen et al. [55]. Neither the affected kindred nor the study population sizes could be retrieved from the majority of the revised papers.

Interestingly, the *ENG* c.277C>T (p.Arg93*) was detected in 12 patients from five unrelated families from various locations in Southeastern Norway [48]. This would have been a grade III founder effect by our grading system, but subsequent haplotype analysis showed different haplotypes and the authors reported it as a hot-spot. Thus, we re-classified it as a repetitive mutation (grade I). Though the c.277C>T variant is reported by a number of authors, confirming the hot-spot [29,44,49,52,59,86], its clustering with distinct haplotypes within a geographical area is surprising. This could have occurred in any of the cases categorized as an even grade III variant merely upon the shared area of ancestry or residence.

## 6. Conclusions

Although HHT is inherited in an autosomal dominant trait and present population isolates with HHT are not known, some causative *ACVRL1* and *ENG* variants are reported with variable evidences for founder effects. This might be attributable to the age-related penetrance, without a significant effect on reproductive fitness. Being aware of local founder variants might simplify HHT gene testing in specific populations, with some potential pitfalls. Large founding kindreds might be potential targets for genetic second-hit studies.

## Figures and Tables

**Table 1:** Our grading system to assess evidences for founder effects in HHT.

Grade	Description
**I**	Identical mutation in apparently unrelated families in a HHT center
**II**	Shared area of ancestry/residence (2–4) OR Shared core haplotype (2–4) OR Genealogical evidence of common ancestry (2–4)
**III**	Shared area of ancestry/residence (≥5) OR Shared core haplotype (≥5) OR Genealogical evidence of common ancestry (≥5) OR shared core haplotype (2–4) AND shared area of ancestry/residence (2–4)
**IV**	Grade 3 AND a locally still-prevalent mutation

Numbers in parentheses refer to the number of families.

**Table 2:** Grade I to IV founder variants in the *ACVRL1* and *ENG* genes.

Location	*ACVRL1* Variant	Type	Classi- Fication	Population	No. of Families	Founder Grade	Comment	Reference	Independent Reference
Ex 3	c.152G>A, p.Cys51Tyr	M	P	Italian (Pavia–-Crema Center)	2	**II**	Shared area of ancestry	[29]	[31,32]
	c.199C>T, p.Arg67Trp	M	P	Italian (Pavia–Crema Center)	2	**III**	Shared haplotype AND area of ancestry	[33]	[34,35,36,37]
					2	II	Shared area of ancestry	[29]	
				German	2	I	Recurrent	[35]	
	c.200G>A, p.Arg67Gln	M	P	French and Italian	3	**II**	Shared haplotype 1	[25]	[38,39]
				French	2	**II**	Shared haplotype 2	[25]	
				Italian (Pavia–Crema Center)	3	**II**	Shared area of ancestry	[29]	
				Italian (Bari Center)	4	I	Recurrent	[31]	
					3	I	Recurrent	[40]	
				Han Chinese	2	I	Recurrent	[41]	
	c.205_209dupTGCGG p.Asn71Alafs*53	FS	P	Italian	2	**II**	Shared area of ancestry	[29]	
	c.265 T>C, p.Cys89Arg	M	LP	Hungarian (Nógrád County)	3	**II ^2^**	Genealogy	[42]	
	289-294delCACAAC p.His97_Asn98del	D	LP ^1^	Italian (Pavia–Crema Center)	2	III	Shared haplotype AND area of ancestry	[33]	
				Italian (Bergamo County)	10	**III-IV ^2^**	Shared area of residence. Prevalent? ^4^	[29]	
Ex 4	c.430C>T, p.Arg144*	N	P	French	2	II	Shared haplotype	[24]	[31,41,43,44,45]
				French and Italian	7	**III ^2^**	Shared haplotype. Age estimate: 22 gen	[25]	
				Italian	4	I	Recurrent	[29]	
Int 5	c.625+1G>C	SS	P	Hungarian (Heves County)	7	**IV ^2^**	Shared area of residence, shared haplotype, genealogy, prevalent ^4^	[46,47]	
Ex 6	c.651G>A, p.Trp217*	N	P	Norwegian (Østfold County and West Sweden)	7	**IV ^2^**	Shared area of ancestry, shared haplotype, prevalent ^4^	[48]	[49]
Ex 7	c.830C>A, p.Thr277Lys	M	P	Norwegian (Rana, Nordland County)	13	**IV ^2^**	Shared area of ancestry, shared haplotype, prevalent ^4^	[48]	
	c.924C>A, p.Cys308*	N	P	Italian (Pavia–Crema Center)	2	**II**	Shared area of ancestry	[29]	[38,40]
	c.998G>T, p.Ser333Ile	M	P	American (Utah, US)	5	**III-IV**	Genealogy. Prevalent? ^4^	[50]	[40]
				American (Toronto Center)	+1		Area of ancestry in Utah, US^4^	[43,51]	
	c.1042delG, p.Asp348Thrfs*6	FS	P	Dutch	7	**III ^2^**	Genealogy in 5 families ^4^	[52]	
Ex 8	c.1055C>A, p.Ala352Asp	M	P	American (Massachusetts, US)	2	**II ^2^**	Shared haplotype	[53]	
**Location**	***ACVRL1* Variant**	**Type**	**Classi-** **Fication**	**Population**	**No. of Families**	**Founder** **Grade**	**Comment**	**Reference**	**Independent** **Reference**
Ex 8	c.1112_1113dupG p.Thr372Hisfs*20	FS	P	French (Valserine Valley, Jura County)	17	**IV ^2^**	Shared area of ancestry/residence, shared haplotype, prevalent ^4^	[24]	
				French	35	**IV ^2^**	Shared area of ancestry/residence, shared haplotype, prevalent. Age estimate: 13 gen ^4^	[25]	
				European and North American	+1?		Area of ancestry in the Rhône-Alpes region, France ^4^	[51]	
				American (Utah, US)	+1		Area of ancestry in Ain, France ^4^	[54]	
	c.1120C>T, p.Arg374Trp	M	P	French and Italian	6	**III ^2^**	Shared haplotype. Age estimate: 11 gen ^4^	[25]	[35,40,55,56,57,58]
				American (Ontario, Canada)	2	I	Recurrent	[34,43]	
				Dutch	3	I	Recurrent	[52]	
				American	2	I	Recurrent	[54]	
				Italian (Bari Center)	2	I	Recurrent	[38]	
				Italian (Pavia–Crema Center)	3	II	Shared area of ancestry	[29]	
				Han Chinese	2	I ^3^	Recurrent	[39]	
	c.1121G>A, p.Arg374Gln	M	P	French (Deux-Sevres County)	3	**III ^2^**	Shared haplotype 1 AND area of ancestry ^4^	[25]	[29,37,56,59,60]
				European and North American	+3?	II	Shared area of ancestry in Parthenay, Deux-Sevres County, France. Age estimate: 4 gen ^4^	[51]	
				French (Northeast France)	3	**III ^2^**	Shared haplotype 2 AND area of ancestry ^4^	[25]	
	c.1126A>G, p.Met376Val	M	P	French	3	**II**	Shared haplotype	[25]	[59]
	c.1199C>A, p.Ala400Asp	M	P	Italian (Pavia–Crema Center)	2	**II**	Shared area of ancestry	[29]	
	c.1231C>T, p.Arg411Trp	M	P	French	7	**III ^2^**	Shared haplotype	[24]	[35,61]
					9	**III ^2^**	Shared haplotype. Age estimate: 15 gen	[25]	
				American (Ontario, Canada)	2	I	Recurrent	[34]	
				Dutch	2	I	Recurrent	[52]	
				German	3	I	Recurrent	[41]	
	c.1232G>A, p.Arg411Gln	M	P	North American	2	**II**	Shared area of ancestry	[51]	[38,40,56,60,62]
				French	2	**II**	Shared haplotype	[25]	
				American (Utah, US)	2	I	Recurrent	[54]	
				Italian (Pavia–Crema Center)	2	**II**	Shared area of ancestry	[29]	
				Han Chinese	3	I ^3^	Recurrent	[39]	
	c.1232G>C, p.Arg411Pro	M	P	French	2	**II**	Shared haplotype	[24]	[59]
**Location**	***ACVRL1* Variant**	**Type**	**Classi-** **Fication**	**Population**	**No. of Families**	**Founder** **Grade**	**Comment**	**Reference**	**Independent** **Reference**
Ex 9	c.1280A>T, p.Asp427Val	M	P ^1^	French	2	**II**	Shared haplotype	[25]	[38]
Int 9	c.1377+2T>A	SS	LP	Hungarian (Heves and Borsod Counties)	2	**II**	Genealogy	[42]	
Ex 10	c.1435C>T, p.Arg479*	N	P	French	2	**II**	Shared haplotype	[24]	[29,37,38,52,59,60,63,64]
					2	**II**	Shared haplotype	[25]	
				Japanese (West Japan)	2	I	Recurrent	[45]	
	c.1450C>T, p.Arg484Trp	M	P	Norwegian (Nordland County)	5	**III-IV ^2^**	Shared haplotype. Prevalent? ^4^	[48]	[25,29,52,61]
				Italian (Bari Center)	2	I	Recurrent	[38]	
**Location**	***ENG* Variant**	**Type**	**Classi-** **Fication**	**Population**	**No. of Families**	**Founder** **Grade**	**Comment**	**Reference**	**Independent** **Reference**
Int 1	c.67+1G>A	SS	P	Netherlands Antillean	7	**IV ^2^**	Shared haplotype, prevalent ^4^	[23]	
				Dutch	+1			[52]	
Ex 3	c.277C>T, p.Arg93*	N	P	Italian	3	**II**	Shared area of ancestry	[29]	[44,52,59,65]
				Norwegian (Southeast)	5	I	Recurrent. Haplotype analysis showed different haplotypes ^4^	[48]	
	c.360C>A, p.Tyr120*	N	P	Danish (Funen County)	7	**IV ^2^**	shared haplotype, shared area of residence, prevalent. Age estimate: 13–14 gen ^4^	[44]	
				Danish (Funen county)	7	**IV ^2^**	Shared area of residence, prevalent ^4^	[66]	
				Danish (Nationwide)	13	**IV ^2^**	Shared area of residence, prevalent ^4^	[67]	
Int 3	c.360+1G>A	SS	P	Italian (Pavia–Crema Center)	2	**II**	Shared area of ancestry	[29]	[41,52,54,68,69]
Ex 6	c.781T>C, p.Trp261Arg	M	P ^1^	Dutch	8	**II**	Genealogy in 3 families ^4^	[52]	
Int 6	c.817-2 A>C	SS	P	Hungarian (Heves and Borsod Counties)	2	**II ^2^**	Shared area of ancestry, genealogy ^4^	[42]	
Ex 7	c.828_829insA, p.Tyr277Ilefs*57	FS	P	Japanese (Akita, County A, Japan)	2	**III-IV ^2^**	Shared haplotype AND area of residence. Prevalent? ^4^	[70]	
Int 7	c.1134+1G>A	SS	P	English (South England)	2	**III**	Shared haplotype AND area of residence	[71]	
Ex 9	c.1238G>T, p.Gly413Val	M	P ^1^	Netherlands Antillean and Dutch	3	**III ^2^**	Shared haplotype AND area of ancestry ^4^	[23]	
				Dutch	+1?			[52]	
Int 10	c.1311G>A, p.Arg437Arg	SS	VUS ^1^	Dutch	5	**III ^2^**	Genealogy ^4^	[52]	
Ex 12	c.1630delA, p.Thr544Profs*8	FS	P	American (Ontario, Canada)	2	**II**	Shared area of ancestry	[34]	
Int 12	c.1686+5G>C	SS	LP ^1^	Spanish	3	**I ^3^**	Recurrent	[60]	

Abbreviations and legends: At the intragenic location, Ex = exon and Int = intron. At the variant type (type), D = in-frame deletion, FS = frameshift, M = missense, N = nonsense and SS = splice-site. At the pathogenicity classification (Classification), P = pathogenic, LP = likely pathogenic, VUS = variant of uncertain significance. ^1^ Pathogenicity of a pending variant is reassessed (Supplementary Materials Table S2). At the founder grade, ^2^ variant reported as a founder; ^3^ variant reported as a founder or a hot-spot. Bolds: the highest founder grade of a variant reported by a given research group. At the comment, gen = generation; ^4^ detailed in the text. Independent Reference: report of the variant by independent authors (if a research group reported the variant several times, the first report is given).

## Supplementary Materials

The following are available online at https://www.mdpi.com/article/10.3390/jcm10081682/s1. Figure S1: Haplotype analysis of patients and unaffected individuals in seven families with the *ACVRL1* c.625+1G>C mutation [46]. Figure S2: Common ancestry of two families exhibiting the ENG c.817-2A>C mutation [42]. Table S1: Publications corresponding to any of the terms “*founder*” and “*founding*” and the etymon “*ancest*”. Table S2: Reassessment of pathogenicity of variants reported as “pending” in the *ENG* and *ACVRL1* Databases [26,27].
