# Supplementary material for: Founder Effects in Hereditary Hemorrhagic Telangiectasia
Source: J Clin Med. 2021 Apr 14;10(8):1682. doi: 10.3390/jcm10081682 (PMC8070971; doi:10.3390/jcm10081682)
Supplement: Supplementary file 1 [file jcm-10-01682-s001.zip › Figure S1_Major et al.pdf]

Figure S1. Haplotype analysis of patients and unaffected individuals in 7 families with the *ACVRL1* c.625+1G>C mutation [46].

	Fam. 1		Family 2																				Family 3							
	● 79		● 82†		□ 62		■ 61		○ 60*		□ 59		□ 36		■ 34		○ 33*		● 5		■ 1		□ 38*		● 37		■ 12		● 9	
D12S85	19	12	19	12	19	18	12	12	12	18	19	18	12	18	12⁺	12	12	12	12⁺	12	12	12	12	12	12	12	12	12	12	12
D12S2196	9	10	10	10	10	12	7	10	10	10	10	12	7	12	7⁺	10	10	10	7⁺	10	10	10	4	10	12	10	11	10	12	10
**rs2071219**	**A**	**G**	**A**	**G**	**A**	**G**	**G**	**G**	**G**	**G**	**A**	**G**	**G**	**G**	**G**	**G**	**A**	**G**	**G**	**G**	**A**	**G**	**A**	**A**	**A**	**G**	**A**	**G**	**A**	**G**
**c.625+1**	**G**	**C**	**G**	**C**	**G**	**G**	**G**	**C**	**G**	**G**	**G**	**G**	**G**	**G**	**G**	**C**	**G**	**G**	**G**	**C**	**G**	**C**	**G**	**G**	**G**	**C**	**G**	**C**	**G**	**C**
**rs706815**	**T**	**T**	**C**	**T**	**C**	**T**	**T**	**T**	**T**	**T**	**C**	**T**	**T**	**T**	**T**	**T**	**T**	**T**	**T**	**T**	**T**	**T**	**T**	**T**	**T**	**T**	**T**	**T**	**T**	**T**
**rs706816**	**A**	**A**	**G**	**A**	**G**	**A**	**A**	**A**	**A**	**A**	**G**	**A**	**A**	**A**	**A**	**A**	**A**	**A**	**A**	**A**	**A**	**A**	**A**	**A**	**A**	**A**	**A**	**A**	**A**	**A**
**D12S1677**	**20**	**20**	**21**	**20**	**21**	**20**	**19**	**20**	**21**	**18**	**21**	**20**	**19**	**20**	**21**	**20**	**20**	**20**	**20**	**20**	**20**	**20**	**19**	**20**	**21**	**20**	**21**	**20**	**20**	**20**
D12S1712	17	16	13	16	13	13	17	16	18	13	13	13	17	13	18	16	25	25	25	16	16	16	16	16	25	16	25	16	8	16
D12S270	19	21	19	21	19	11	11	21	11	11	19	11	11	11	11	21	11	11	11	21	11	21	11	21	19	21	19	21	19	21

	Family 4										Family 5																Family 6						Family 7			
	□ 58		■ 56		○ 54*		■ 37		■ 33		● 67		○ 66		□ 64		● 56		□ 53*		● 33		○ 30		○ 1		■ 67		● 61		■ 38		● 68		● 65	
D12S85	12	12	12	12	20	12	20	12	20	12	12	12	12	19	12	19	18	12	12	20	18•	12	18	12	12	20	17	12	12	12	12	12	20	12	20	12
D12S2196	10	11	7	10	11	10	11	10	11	10	11	10	10	7	10	7	7	10	10	7	7•	10	7	10	7	11	11	10	10	10	11	10	10	10		
**rs2071219**	**A**	**A**	**A**	**G**	**A**	**G**	**A**	**G**	**A**	**G**	**A**	**G**	**A**	**G**	**A**	**G**	**G**	**G**	**G**	**A**	**G**	**G**	**G**	**G**	**A**	**G**	**G**	**G**	**G**	**G**	**A**	**G**	**G**	**G**	**A**	**G**
**c.625+1**	**G**	**G**	**G**	**C**	**G**	**G**	**G**	**C**	**G**	**C**	**G**	**C**	**G**	**G**	**G**	**G**	**G**	**C**	**G**	**G**	**G**	**C**	**G**	**G**	**G**	**G**	**G**	**C**	**G**	**C**	**G**	**C**	**G**	**C**	**G**	**C**
**rs706815**	**T**	**C**	**C**	**T**	**C**	**T**	**C**	**T**	**C**	**T**	**C**	**T**	**T**	**T**	**T**	**T**	**T**	**T**	**T**	**C**	**T**	**T**	**T**	**T**	**T**	**T**	**T**	**T**	**T**	**T**	**T**	**T**	**T**	**T**	**C**	**T**
**rs706816**	**A**	**G**	**G**	**A**	**G**	**A**	**G**	**A**	**G**	**A**	**G**	**A**	**A**	**A**	**A**	**A**	**A**	**A**	**A**	**G**	**A**	**A**	**A**	**A**	**A**	**A**	**A**	**A**	**A**	**A**	**A**	**A**	**A**	**A**	**G**	**A**
**D12S1677**	**20**	**21**	**21**	**20**	**21**	**21**	**21**	**20**	**21**	**20**	**22**	**20**	**18**	**20**	**18**	**20**	**20**	**20**	**18**	**21**	**18**	**20**	**20**	**18**	**18**	**20**	**19**	**20**	**19**	**20**	**20**	**20**	**19**	**20**		
D12S1712	16	17	18	16	16	16	16	16	16	16	17	16	13	17	13	17	24	16	16	15	16	16	24	16	16	18	17	16	17	16	17	16	17	16		
D12S270	11	11	21	21	11	9	11	21	11	21	11	21	11	11	11	11	11	21	9	11	9	21	11	9	9	19	9	21	9	9	11	11	11	11		

Legends: blackened individuals are heterozygous, individual age at haplotype analysis is indicated; †: patient died in the year of haplotype analysis; *: non-consanguineous spouses; •: haplotype modification probably caused by recombination; intragenic polymorphic markers are highlighted with bold characters and the mutation site is additionally underscored; chromosomal location of the STR sequences: D12S85 $(AC)_n$: 12q13.11 (47,336,687-47,336,862); D12S2196 $(CTAT)_n$: 12q13.11 (48,700,248-48,700,547); D12S1677 $(GT)_n$: 12q13.13 (53,312,927-53,313,286); D12S1712 $(GT)_n$: 12q13.13 (52,445,897-52,446,222); D12S270 $(CA)_n$: 12q13.13 (52,709,971-52,710,294). Chromosomal location of the *ACVRL1* intragenic SNPs: rs2071219 (c.314-35A>G, MAF 0.437), rs706815 (c.1377+45T>C, MAF 0.343) and rs706816 (c.1377+65A>G, MAF 0.343); in the case of a 65-year-old female in Family 7 haplotype analysis was incomplete.
