# Supplementary material for: Founder Effects in Hereditary Hemorrhagic Telangiectasia
Source: J Clin Med. 2021 Apr 14;10(8):1682. doi: 10.3390/jcm10081682 (PMC8070971; doi:10.3390/jcm10081682)
Supplement: Supplementary file 1 [file jcm-10-01682-s001.zip › Figure S2_Major et al.pdf]

Figure S2. Common ancestry of two families exhibiting the *ENG* c.817-2A>C mutation [42].

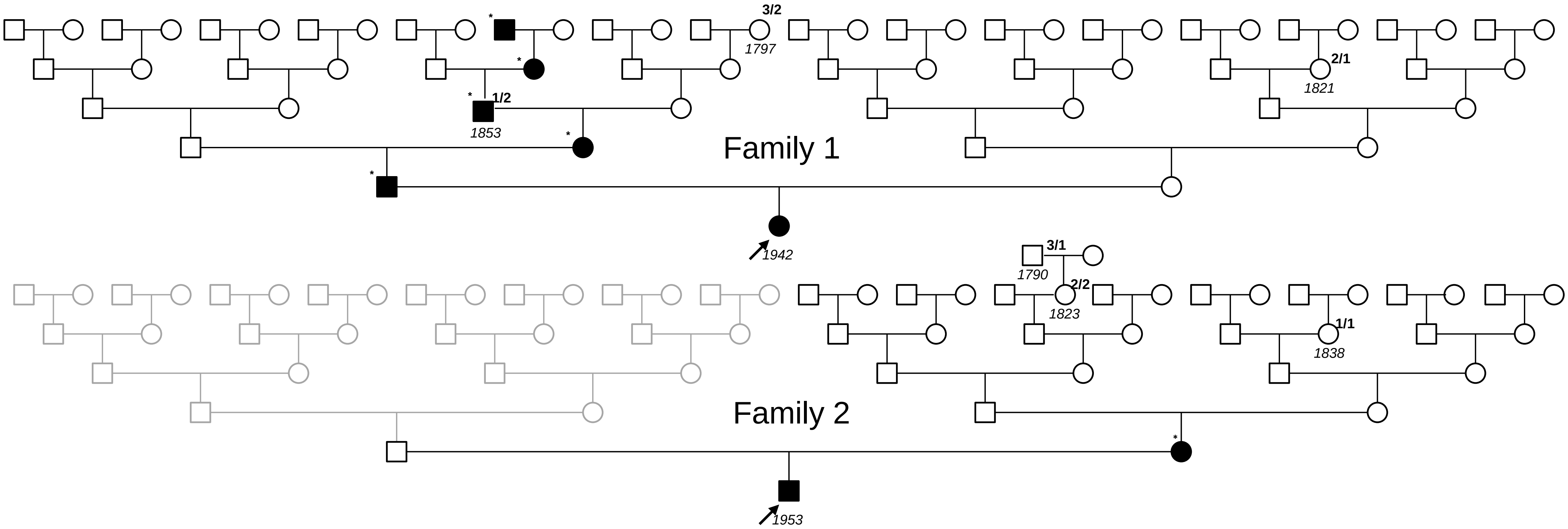

Legends: The genealogical trees based on parish registries. Probands with definite HHT are marked with arrows. Blackened individuals are affected; *: deceased individual with epistaxis and telangiectases by hearsay; individuals designated with 1/1-1/2, 2/1-2/2 and 3/1-3/2 respectively, are siblings; birth years of individuals are in italics. The paternal branch of Family 2 proband is unknown. Based on information by hearsay, HHT might derive from siblings 1/1-1/2
