# Supplementary material for: Founder Effects in Hereditary Hemorrhagic Telangiectasia
Source: J Clin Med. 2021 Apr 14;10(8):1682. doi: 10.3390/jcm10081682 (PMC8070971; doi:10.3390/jcm10081682)
Supplement: Supplementary file 1 [file jcm-10-01682-s001.zip › Table S1_Major et al.pdf]

Table S1. Publications corresponding to any of the terms "*founder*", "*founding*" and the etymon "*ancest*".

	**Publication**	**Ref No.**
1.	Abdalla, S. A.; Geisthoff, U. W.; Bonneau, D. ; Plauchu, H. ; McDonald, J. ; Kennedy, S. ; Faughnan, M. E. ; Letarte, M. Visceral manifestations in hereditary haemorrhagictelangiectasia type 2. *J Med Genet* **2003**, *40*, 494–502. [http://doi.org/10.1136/jmg.40.7.494] [PMID: 12843319]	[51]
2.	Abdalla, S. A.; Cymerman, U. ; Rushlow, D. ; Chen, N. ; Stoeber, G. P.; Lemire, E. G. ; Letarte, M. Novel mutations and polymorphisms in genes causing hereditary hemorrhagic telangiectasia. *Hum Mutat* **2005**, *25*, 320–321. [http://doi.org/10.1002/humu.9312] [PMID: 15712271]	[34]
3.	Albiñana, V.; Zafra, M. P. ; Colau, J. ; Zarrabeitia, R. ; Recio-Poveda, L. ; Olavarrieta, L.; Pérez-Pérez, J.; Botella, L. M. Mutation affecting the proximal promoter of Endoglin as the origin of hereditary hemorrhagic telangiectasia type 1. *BMC Med Genet* **2017**, *18*, 20. [http://doi.org/10.1186/s12881-017-0380-0] [PMID 28231770]	
4.	Alvarez, H.; Castillo, M. Genetic Markers and Their Influence on Cerebrovascular Malformations. *Neuroimag Clin N Am* **2015**, *25*, 69–82. [http://doi.org/10.1016/j.nic.2014.09.009] [PMID: 25476513]	
5.	Bayrak-Toydemir, P.; Mao, R. ; Lewin, S. ; McDonald, J. Hereditary hemorrhagic telangiectasia: An overview of diagnosis and management in the molecular era for clinicians. *Genet Med* **2004**, *6*, 175–191. [http://doi.org/10.1097/01.gim.0000132689.25644.7c]	
6.	Bayrak-Toydemir, P.; McDonald, J.; Markewitz, B.; Lewin, S.; Miller, F.; Chou, L.; Gedge, F.; Tang, W.; Coon, H.; Mao, R. Genotype-phenotype correlation in hereditary hemorrhagic telangiectasia: mutations and manifestations. *Am J Med Genet* **2006**, *140*, 463-470. [http://doi.org/10.1002/ajmg.a.31101] [PMID: 16470787]	[54]
7.	Benzinou, M.; Clermont, F. F.; Letteboer, T. G. W.; Kim, J.; Espejel, S.; Harradine, K. A.; Arbelaez, J.; Luu, T.; Roy, R.; Quigley, D.; Higgins, M., N.; Zaid, M.; Aouizerat, B. E.; van Amstel, J., K., P.; Giraud, S.; Dupuis-Girod, S.; Lesca, G.; Plauchu, H.; Hughes, C. C. W.; Westermann, C., J., J.; Akhurst, R. J. Mouse and human strategies identify PTPN14 as a modifier of angiogenesis and hereditary haemorrhagic telangiectasia. *Nature Communications* **2012**, *3*. [http://doi.org/10.1038/ncomms1633] [PMID: 22233626]	
8.	Bideau, A.; Brunet, G.; Heyer, E.; Plauchu, H.; Robert, J.-M. An abnormal concentration of cases of Rendu-Osler disease in the Valserine valley of the French Jura: a genealogical and demographic study. *Ann Hum Biol* **1992**, *19, 233–247.* [http://doi.org/10.1080/03014469200002112] [PMID: 1616282]	
9.	Boeri, L.; Radi, O.; Canzonieri, C.; Buscarini, E.; Scatigno, A.; Minelli, A.; Ornati, F.; Pagella, F.; Danesino, C.; Olivieri, C. Hereditary Hemorrhagic Telangiectasia: Breakpoint characterization of a novel large deletion in ACVRL1 suggests the causing mechanism. *Mol Syndromol* **2013**, *4*, 119–124. [http://doi.org/10.1159/000347029] [PMID:]	
10.	Brunet, G.; Lesca, G.; Génin, E.; Dupuis-Girod, S.; Bideau, A.; Plauchu, H. Thirty Years of Research into Rendu-Osler-Weber Disease in France: Historical Demography, Population Genetics and Molecular Biology. *Population* **2009**, 273-291. [http://doi.org/10.3917/pope.902.0273]	[74]
11.	Brusgaard, K.; Kjeldsen, A.; Poulsen, L.; Moss, H.; Vase, P.; Rasmussen, K.; Kruse T. A.; Hørder, M. Mutations in endoglin and in activin receptor-like kinase 1 among Danish patients with hereditary haemorrhagic telangiectasia. *Clin Genet* **2004**, *66*, 556–561. [http://doi.org/10.1111/j.1399-0004.2004.00341.x] [PMID: 15521985]	[44]
12.	Canzonieri, C.; Ornati, F.; Matti, E.; Chu, F.; Manfredi, G.; Olivieri, C.; Buscarini, E.; Pagella, F. Hereditary haemorrhagic telangiectasia in North African and sub-Saharan patients. *S Afr Med J* **2014**, *104*, 256-257. [http://doi.org/10.7196/samj.7816] [PMID: 25118534]	[76]

13. Cartin-Ceba, R.; Swanson, K. L.; Krowka, M. J. Pulmonary Arteriovenous Malformations. *Chest* **2013**, *144,* 1033–1044. [http://doi.org/10.1378/chest.12-0924] [PMID: 24008954]

14. Caselitz, M.; Wagner, S.; Manns, M. P. Liver Involvement in Osler–Weber–Rendu Disease (Hereditary Hemorrhagic Telangiectasia, HHT). In *Zakim and Boyer's Hepatology,* 5th ed. Boyer, T., Wright, T., Manns, M., Eds.; Saunders: Canada, 2006; Volume 1, pp. 915–929. [http://doi.org/10.1016/b978-1-4160-3258-8.50053-x]

15. Chen, Y.-J.; Yang, Q.-H.; Liu, D.; Liu, Q.-Q.; Eyries, M.; Wen, L.; Jiang, X.; Yuan, P.; Zhang, R.; [37]
Soubrier, F.; Jing, Z.-C. Clinical and genetic characteristics of Chinese patients with hereditary haemorrhagic telangiectasia-associated pulmonary hypertension. *Eur J Clin Invest* **2013**, *43,* 1016–1024. [http://doi.org/10.1111/eci.12138] [PMID: 23919827]

16. Cohen, J. H.; Faughnan, M. E.; Letarte, M.; Vandezande, K.; Kennedy, S. J.; Krahn, M. D. Cost comparison of genetic and clinical screening in families with hereditary hemorrhagic telangiectasia. *Am J Med Genet* **2005**, *137,* 153–160. [http://doi.org/10.1002/ajmg.a.30838] [PMID: 16059938]

17. Cymerman, U.; Vera, S.; Karabegovic, A.; Abdalla, S.; Letarte, M. Characterization of 17 novel endoglin mutations associated with hereditary hemorrhagic telangiectasia. *Human Mutation* **2003**, *21*, 482–492. [http://doi.org/10.1002/humu.10203]

18. Dakeishi, M.; Shioya, T.; Wada, Y.; Shindo, T.; Otaka, K.; Manabe, M.; Nozaki, J.; Inoue, S.; [70]
Koizumi, A. Genetic epidemiology of hereditary hemorrhagic telangiectasia in a local community in the northern part of Japan. *Hum Mutat* **2002**, *19,* 140–148. [http://doi.org/10.1002/humu.10026] [PMID: 11793473]

19. Damjanovich, K.; Langa, C.; Blanco, F. J.; McDonald, J.; Botella, L. M.; Bernabeu, C.; Wooderchak-Donahue, W.; Stevenson, D. A.; Bayrak-Toydemir, P. 5′UTR mutations of ENG cause Hereditary Hemorrhagic Telangiectasia. *Orphanet J Rare Dis* **2011**, *6, 85.* [http://doi.org/10.1186/1750-1172-6-85] [PMID: 22192717]

20. Dupuis-Girod, S.; Bailly, S.; Plauchu, H. Hereditary hemorrhagic telangiectasia: from molecular biology to patient care. *J Thromb Haemost* **2010**, *8,* 1447–1456. [http://doi.org/10.1111/j.1538-7836.2010.03860.x] [PMID: 20345718]

21. Giraud, S.; Bardel, C.; Dupuis-Girod, S.; Carette, M.-F.; Gilbert-Dussardier, B.; Riviere, S.; Jean-Christophe Saurin, J.-C.; Eyries, M.; Patri, S.; Decullier, E.; Calender, A.; Lesca, G. Sequence variations of ACVRL1 play a critical role in hepatic vascular malformations in hereditary hemorrhagic telangiectasia. *Orphanet J Rare Dis* **2020**, *15.* [http://doi:10.1186/s13023-020-01533-2] [PMID: 32962750]

22. Fernández-Jorge, B.; Losada, J. D. P.; Paradela, S.; Martínez-González, C.; Fonseca, E. Treatment of cutaneous and mucosal telangiectases in hereditary hemorrhagic telangiectasia: Report of three cases. *J Cosmet Laser Ther* **2007**, *9,* 29–33. [http://doi.org/10.1080/14764170601091687] [PMID: 17506138]

23. Fontalba, A.; Fernandez-L, A.; García-Alegria, E., Albiñana, V.; Garrido-Martin, E. M.; Blanco, F. [60]
J.; Zarrabeitia, R.; Perez-Molino, A.; Bernabeu-Herrero, M. E.; Ojeda M.-L.; Fernandez-Luna, J. L.; Bernabeu, C.; Botella, L. M. Mutation study of Spanish patients with Hereditary Hemorrhagic Telangiectasia. *BMC Med Genet* **2008**, *9.* [http://doi.org/10.1186/1471-2350-9-75] [PMID: 18673552]

24. Gallione, C. J.; Scheessele, E. A.; Reinhardt, D.; Duits, A. J.; Berg, J. N.; Westermann, C. J. J.; [23]
Marchuk, D. A. Two common endoglin mutations in families with hereditary hemorrhagic telangiectasia in the Netherlands Antilles: evidence for a founder effect. *Hum Genet* **2000**, *107, 40–44.* [http://doi.org/10.1007/s004390000326] [PMID: 10982033]

25. Gariballa, N.; Ali, B. R. Endoplasmic Reticulum Associated Protein Degradation (ERAD) in the Pathology of Diseases Related to TGFβ Signaling Pathway: Future Therapeutic Perspectives. *Front Mol Biosci* **2020**, *7.* [http://doi.org/10.3389/fmolb.2020.575608] [PMID: 33195419]

26. Giordano, P.; Nigro, A.; Lenato, G. M.; Guanti, G.; Suppressa, P.; Lastella, P.; De Mattia, D.; Sabba, C. Screening for children from families with Rendu-Osler-Weber disease: from geneticist to clinician. *J Thromb Haemost* **2006**, *4, 1237–1245.* [http://doi.org/10.1111/j.1538-7836.2006.01934.x] **[**PMID: 16706966**]** [31]

27. Harrison, R. E.; Flanagan, J. A.; Sankelo, M.; Abdalla, S. A.; Rowell, J.; Machado, R. D.; Elliott, C. G.; Robbins, I. M.; Olschewski, H.; McLaughlin, V.; Gruenig, E.; Kermeen, F.; Laitinen, T.; Morrell, N W.; Trembath, R. C. Molecular and functional analysis identifies ALK-1 as the predominant cause of pulmonary hypertension related to hereditary haemorrhagic telangiectasia. *J Med Genet* **2003**, *40,* 865–871. [http://doi.org/10.1136/jmg.40.12.865] **[**PMID: 14684682**]** [56]

28. Heimdal, K.; Dalhus, B.; Rødningen, O. K.; Kroken, M.; Eiklid, K.; Dheyauldeen, S.; Røysland T.; Andersen R.; Kulseth, M. A. Mutation analysis in Norwegian families with hereditary hemorrhagic telangiectasia: founder mutations inACVRL1. *Clinical Genetics* **2015**, *89,* 182–186. [http://doi:10.1111/cge.12612] **[**PMID: 25970827**]** [48]

29. Kjeldsen, A. D.; Moller, T. R.; Brusgaard, K.; Vase, P.; Andersen, P. E. Clinical symptoms according to genotype amongst patients with hereditary haemorrhagic telangiectasia. *J Intern Med* **2005**, *258,* 349–355. [http://doi.org/10.1111/j.1365-2796.2005.01555.x] **[**PMID: 16164574**]** [66]

30. Lee, S.-T.; Kim, J.-A.; Jang, S.-Y.; Kim, D.-K.; Do, Y. S.; Suh, G. Y.; Kim, J.-W.; Ki, C.-S. Clinical Features and Mutations in the ENG, ACVRL1, and SMAD4 genes in Korean Patients with Hereditary Hemorrhagic Telangiectasia. *J Korean Med Sci* **2009**, *24,* 69. [http://doi.org/10.3346/jkms.2009.24.1.69] [PMID: 19270816]

31. Lee, N. P.; Matevski, D.; Dumitru, D.; Piovesan, B.; Rushlow, D.; Gallie, B. L. Identification of clinically relevant mosaicism in type I hereditary haemorrhagic telangiectasia. *J Med Genet* **2011**, *48,* 353–357. [http:/doi.org/10.1136/jmg.2010.088112] [PMID: 21415079]

32. Lenato, G. M.; Lastella, P.; Di Giacomo, M. C.; Resta, N.; Suppressa, P.; Pasculli, G.; Sabbà, C.; Guanti, G. DHPLC-based mutation analysis of ENG and ALK-1 genes in HHT Italian population. *Hum Mutat* **2006**, *27,* 213–214. [http://doi.org/10.1002/humu.9400] **[**PMID: 16429404**]** [38]

33. Lesca, G.; Plauchu, H.; Coulet, F.; Lefebvre, S.; Plessis, G.; Odent, S.; Rivière, S.; Leheup, B.; Goizet,C.; Carette, M.-F.; Cordier, J.-F.; Pinson, S.; Soubrier, F.; Calender, A.; Giraud, S. Molecular screening ofALK1/ACVRL1andENGgenes in hereditary hemorrhagic telangiectasia in France. *Hum Mutat* **2004**, *23,* 289–299. [http://doi.org/10.1002/humu.20017] **[**PMID: 15024723**]** [24]

34. Lesca, G.; Burnichon, N.; Raux, G.; Tosi, M.; Pinson, S.; Marion, M. J.; Babin, E.; Gilbert-Dussardier, B.; Rivière, S.; Goizet, C.; Faivre, L.; Plauchu, H.; Frébourg, T.; Calender, A.; Giraud, S.; French Rendu-Osler Network. Distribution of ENG and ACVRL1 (ALK1) mutations in French HHT patients. *Hum Mutat* **2006**, *27,* 598. [http://doi.org/10.1002/humu.9421] [PMID: 16705692] [65]

35. Lesca, G.; Genin, E.; Blachier, C.; Olivieri, C.; Coulet, F.; Brunet, G.; Dupuis-Girod, S.; Buscarini, E.; Soubrier, F.; Calender, A.; Danesino, C.; Giraud, S.; Plauchu, H. Hereditary hemorrhagic telangiectasia: evidence for regional founder effects of ACVRL1 mutations in French and Italian patients. *Eur J Hum Genet* **2008**, *16,* 742–749. [doi:10.1038/ejhg.2008.3] **[**PMID: 18285823**]** [25]

36. Letteboer, T. G. W.; Zewald, R. A.; Kamping, E. J.; de Haas, G.; Mager, J. J.; Snijder, R. J.; Lindhout, D.; Hennekam, F. A. M.; Westermann, C. J. J.; Ploos van Amstel, J. K. Hereditary hemorrhagic telangiectasia: ENG and ALK-1 mutations in Dutch patients. *Hum Genet* **2004**, *116,* 8–16. [http://doi.org/10.1007/s00439-004-1196-5] **[**PMID: 15517393**]** [52]

37. Major, T.; Gindele, R.; Szabó, Z.; Alef, T.; Thiele, B.; Bora, L.; Kis Z.; Bárdossy, P.; Rácz, T.; Havacs, I.; Bereczky, Z. Evidence for the founder effect of a novel ACVRL1 splice-site mutation in Hungarian hereditary hemorrhagic telangiectasia families. *Clin Genet* **2016**, *90,* 466–467. [http://doi.org/10.1111/cge.12806] **[**PMID: 27291782**]** [46]

38. Major, T.; Gindele, R.; Szabó, Z.; Jóni, N.; Kis, Z.; Bora, L.; Bárdossy, P.; Rácz, T.; Karosi, T.; [42]

Bereczky, Z. A herediter haemorrhagiás teleangiectasia (Osler–Weber–Rendu-kór) genetikai diagnosztikája. *Orv Hetil* **2019**, *160,* 710–719. [http://doi.org/10.1556/650.2019.31380] [PMID: 31030535]

39. Major, T.; Gindele, R.; Szabó, Z.; Kis, Z.; Bora, L.; Jóni, N.; Bárdossy, P.; Rácz, T.; Bereczky, Z. The [47]
Stratified Population Screening of Hereditary Hemorrhagic Telangiectasia. *Pathol Oncol Res* **2020**, *26*, 2783-2788. [http://doi.org/10.1007/s12253-019-00602-7] [PMID: 30685840]

40. Marchuk, D. A.; Srinivasan, S.; Squire, T. L.; Zawistowsk, J. S. Vascular morphogenesis: tales of two syndromes. *Hum Mol Genet* 2003, *12*, 97–112. [http://doi.org/10.1093/hmg/ddg103] [PMID: 12668602]

41. Martín-Llahí, M.; Albillos, A.; Bañares, R.; Berzigotti, A.; García-Criado, M. Á.; Genescà, J.; Hernández-Gea, V.; Llop-Herrera, E.; Masnou-Ridaura, H.; Mateok, J.; Navascués, C. A.; Puente, Á.; Romero-Gutiérrez, M.; Simón-Taleroc, M.; Téllez, L.; Turonc, F.; Villanuevac, C.; Zarrabeitia, R.; García-Pagán, J. C. Vascular diseases of the liver. Clinical Guidelines from the Catalan Society of Digestology and the Spanish Association for the Study of the Liver. *Gastroenterol Hepatol* **2017**, *40*, 538-580. [http://doi:10.1016/j.gastrohep.2017.03.011] [PMID: 28610817]

42. Massa, M.; Canzonieri, C.; Campanelli, R.; Ornati, F.; Fois, G.; Pagella, F.; Matti, E.; Buscarini, E.; Danesino, C.; Rosti, V.; Olivieri, C. Increase of circulating endothelial cells in patients with Hereditary Hemorrhagic Telangiectasia. *Int J Hematol* **2014**, *101,* 23–31. [http://doi.org/10.1007/s12185-014-1698-4] [PMID: 25465912]

43. McDonald, J. E.; Miller, F. J.; Hallam, S. E.; Nelson, L.; Marchuk, D. A.; Ward, K. J. Clinical [50]
manifestations in a large hereditary hemorrhagic telangiectasia (HHT) type 2 kindred. *Am J Med Genet* **2000**, *93,* 320–327. [doi: 10.1002/1096-8628(20000814)93:4<320::aid-ajmg12>3.0.co;2-r] [PMID: 10946360]

44. Olivieri, C.; Mira, E.; Delù, G.; Pagella, F.; Zambelli, A.; Malvezzi, L.; Buscarini, E.; Danesino, C. [33]
Identification of 13 new mutations in the ACVRL1 gene in a group of 52 unselected Italian patients affected by hereditary haemorrhagic telangiectasia. *J Med Genet* **2002**, *39,* 39e–39. [http://doi.org/10.1136/jmg.39.7.e39] [PMID: 12114496]

45. Olivieri, C.; Pagella, F.; Semino, L.; Lanzarini, L.; Valacca, C.; Pilotto, A.; Corno, S.; Scappaticci, S.; [29]
Manfredi, G.; Buscarini, E.; Danesino, C. Analysis of ENG and ACVRL1 genes in 137 HHT Italian families identifies 76 different mutations (24 novel). Comparison with other European studies. *J Hum Genet* **2007**, *52*, 820–829. [http://doi.org/10.1007/s10038-007-0187-5] [PMID: 17786384]

46. Paquet, M. E.; Pece-Barbara, N.; Vera, S.; Cymerman, U.; Karabegovic, A.; Shovlin, C.; Letarte, M. Analysis of several endoglin mutants reveals no endogenous mature or secreted protein capable of interfering with normal endoglin function. *Hum Mol Genet* **2001**, *10,* 1347–1357. [http://doi.org/10.1093/hmg/10.13.1347] [PMID: 11440987]

47. Prigoda, N. L.; Savas, S.; Abdalla, S. A.; Piovesan, B.; Rushlow, D.; Vandezande, K.; Zhang, E.; Ozcelik, H.; Gallie, B. L.; Letarte, M. Hereditary haemorrhagic telangiectasia: mutation detection, test sensitivity and novel mutations. *J Med Genet* **2006**, *43,* 722–728. [http://doi.org/10.1136/jmg.2006.042606] [PMID: 16690726]

48. Ricard, N.; Bidart, M.; Mallet, C.; Lesca, G.; Giraud, S.; Prudent, R.; Feige, J.-J.; Bailly, S. Functional [32]
analysis of the BMP9 response of ALK1 mutants from HHT2 patients: a diagnostic tool for novel ACVRL1 mutations. *Blood* **2010**, *116,* 1604–1612. [http://doi.org/10.1182/blood-2010-03-276881] [PMID: 20501893]

49. Robert, F.; Desroches-Castan, A.; Bailly, S.; Dupuis-Girod, S.;Feige, J.-J. Future treatments for hereditary hemorrhagic telangiectasia. *Orphanet J Rare Dis* **2020**, *15.* [http://doi.org/10.1186/s13023-019-1281-4] [PMID: 31910860]

50. Ruiz-Llorente, L.; Gallardo-Vara, E.; Rossi, E.; Smadja, D. M.; Botella, L. M.; Bernabeu, C. Endoglin and alk1 as therapeutic targets for hereditary hemorrhagic telangiectasia. *Expert Opin*

Ther Targets **2017**, *21*, 933–947. [http://doi.org/10.1080/14728222.2017.1365839] [PMID: 28796572]

51. Sabbà, C. A rare and misdiagnosed bleeding disorder: hereditary hemorrhagic telangiectasia. *J Thromb and Haemost* **2005**, *3*, 2201–2210. [http://doi.org/10.1111/j.1538-7836.2005.01345.x] [PMID: 15892861]

52. Sankelo, M.; Halme, M.; Laitinen, T.;Mattila, P. S. Hereditary hemorrhagic telangiectasia type 1 and 2 mutations in Finland. *Acta Otolaryngol* 2008, *128*, 1238–1241. [http://doi.org/10.1080/00016480801908035] [PMID: 18607909]

53. Sell, B.; Evans, J.;Horn, D. Brain Abscess and Hereditary Hemorrhagic Telangiectasia. *South Med J* **2008**, *101*, 618–625. [http://doi.org/10.1097/smj.0b013e318172f716] [PMID: 18475224]

54. Shenkar, R.; Elliott, J. P.; Diener, K.; Gault, J.; Hu, L.-J.; Cohrs, R. J.; Phang, T.; Hunter, L.; Breeze, R. E.; Awad, I. A. Differential Gene Expression in Human Cerebrovascular Malformations. *Neurosurgery* 2003, *52*, 465–78. [http://doi.org/10.1227/01.neu.0000044131.03495.22]

55. Shovlin, C. L.; Hughes, J. M. B.; Scott, J.; Seidman, C. E.; Seidman, J. G. Characterization of [71] Endoglin and Identification of Novel Mutations in Hereditary Hemorrhagic Telangiectasia. *Am J Hum Genet* **1997**, *61*, 68–79. [http://doi.org/10.1086/513906] **[**PMID: 9245986**]**

56. Shovlin, C. L. Hereditary haemorrhagic telangiectasia: Pathophysiology, diagnosis and treatment. *Blood Rev*, **2010**, *24*, 203–219. [http://doi.org/10.1016/j.blre.2010.07.001] [PMID: 20870325]

57. Smoot, L. B.; Obler, D.; McElhinney, D. B.; Boardman, K.; Wu, B.-L.; Lip, V.; Mullen, M. P. Clinical [53] features of pulmonary arterial hypertension in young people with an ALK1 mutation and hereditary haemorrhagic telangiectasia. *Arch Dis Child* **2009**, *94*, 506–511. [http://doi.org/10.1136/adc.2007.133082] **[**PMID: 19357124**]**

58. Song, J.; Eichstaedt, C. A.; Viales, R. R.; Benjamin, N.; Harutyunova, S.; Fischer, C.; Grunig, E.; Hinderhofer, K. Identification of genetic defects in pulmonary arterial hypertension by a new gene panel diagnostic tool. *Clin Sci (Lond)* **2016**, *130*, 2043–2052. [http://doi.org/10.1042/cs20160531] [PMID: 27613157]

59. Steineger, J.; Geirdal, A. Ø.; Osnes, T.; Heimdal, K. R.; Dheyauldeen, S. Intranasal bevacizumab injections improve quality of life in HHT patients. *Laryngoscope* **2019**, *130*, E284-E288. [http://doi.org/10.1002/lary.28179] [PMID: 31287573]

60. Toporsian, M.; Letarte, M. Hereditary Hemorrhagic Telangiectasia: A Model to Probe the Biology of the Vascular Endothelium. *Endothelial Biomedicine* **2007**, 1113–1123. [http://doi.org/10.1017/cbo9780511546198.123]

61. Tørring, P. M., Brusgaard, K., Ousager, L. B., Andersen, P. E., Kjeldsen, A. D. National mutation [67] study among Danish patients with hereditary haemorrhagic telangiectasia. *Clin Genet* **2013**, *86*, 123–133. [http://doi:10.1111/cge.12269] **[**PMID: 24001356**]**

62. Vallon, M.; Chang, J.; Zhang, H.; Kuo, C. J. Developmental and pathological angiogenesis in the central nervous system. *Cell Mol Life Sci* **2014**, *71, 3489–3506.* [http://doi.org/10.1007/s00018-014-1625-0] [PMID: 24760128]

63. van den Driesche, S.; Mummery, C.; Westermann, C. Hereditary hemorrhagic telangiectasia: an update on transforming growth factor ⊚ signaling in vasculogenesis and angiogenesis. *Cardiovasc Res* **2003**, *58*, 20–31. [http://doi.org/10.1016/s0008-6363(02)00852-0]

64. Waite, K. A.; Eng, C. From developmental disorder to heritable cancer: it's all in the BMP/TGF-β family. *Nat Rev Genet* **2003**, *4, 763–773.* [http://doi.org/10.1038/nrg1178] [PMID: 14526373]

65. Wehner, L.-E.; Folz, B.; Argyriou, L.; Twelkemeyer, S.; Teske, U.; Geisthoff, U.; Wernerb, J. A.; [58] Engel, W.; Nayernia, K. Mutation analysis in hereditary haemorrhagic telangiectasia in Germany reveals 11 novel ENG and 12 novel ACVRL1/ALK1 mutations. *Clin Genet* 2006, *69*, 239–245.

[http://doi.org/10.1111/j.1399-0004.2006.00574.x] [PMID: 16542389]

66. Westermann, C. J. J.; Rosina, A. F.; de Vries, V.; de Coteau, P. A. The prevalence and manifestations of hereditary hemorrhagic telangiectasia in the Afro-Caribbean population of the Netherlands Antilles: A family screening. *Am J Med Genet* **2003**, *116,* 324–328. [http://doi.org/10.1002/ajmg.a.10002] **[**PMID: 12522784**]** [75]

67. Zhao, Y.; Zhang, Y.; Wang, X.; Zhang, L. *Variant* analysis in Chinese families with hereditary hemorrhagic telangiectasia. *Mol Genet Genomic Med* **2019**, *7*:e893. [http://doi.org/10.1002/mgg3.893] **[**PMID: 31400083**]** [39]

Abbreviations and legends: Papers are listed in alphabetic order; Ref No.: reference number in the text.
