# Supplementary material for: Founder Effects in Hereditary Hemorrhagic Telangiectasia
Source: J Clin Med. 2021 Apr 14;10(8):1682. doi: 10.3390/jcm10081682 (PMC8070971; doi:10.3390/jcm10081682)
Supplement: Supplementary file 1 [file jcm-10-01682-s001.zip › Table S2_Major et al.pdf]

Table S2. Reassessment of pathogenicity of variants reported as "pending" in the *ENG* and *ACVRL1* Databases [26,27]

Variant	*In silico* predilection				HGMD Accession Number	Authors' report [Ref. No.]	Patogenicity (ACMG)
	PolyPhen2		MutPred2*	SIFT**			
	HumDiv*	HumVar*					
ACVRL1 c.289-294delCACAAC (p.His97_Asn98del)					**CD023489**	Absent in healthy controls (n=50), deletes two highly conserved amino acids of the extracellular domain, compared to ortologs [33]	Likely pathogenic
ACVRL1 c.1280A>T (p.Asp427Val)	probably damaging with a score of 1.000	probably damaging with a score of 0.999	pathogenic (0.820)	AFFECT PROTEIN FUNCTION with a score of 0.00	**CM060792**	Absent in healthy controls (n=100), cosegregated with disease in families, affecting conserved amino acids among ortologs [38]. Absent in healthy controls (n=20) [25]	Pathogenic
ENG c.781T>C (p. Trp261Arg)	probably damaging with a score of 0.999	probably damaging with a score of 0.997	pathogenic (0.863)	AFFECT PROTEIN FUNCTION with a score of 0.00	**CM050044**	"Deleterious" [52]	Pathogenic
ENG c.1238G>T (p. Gly413Val)	probably damaging with a score of 1.000	probably damaging with a score of 1.000	pathogenic (0.763)	AFFECT PROTEIN FUNCTION with a score of 0.00	**CM001137**	Absent in healthy controls (104 North American or northern European whites, 42 Antilleans, 67 Dutch samples), cosegregated with disease in the Antillean and Dutch families exhibiting a shared core haplotype; [23]	Pathogenic
ENG c.1311G>A (p. Arg437Arg)	-	-	-	TOLERATED with a score of 1.00	-	Silent mutation occuring at the last position of exon9b . This position is part of the consensus sequence of the donor splice site and is highly conserved [52]	VUS
ENG c.1686+5G>C					**CS085778**	Absent in healthy controls (n=100), cosegregated with disease in families [60]	Likely pathogenic

Abbreviations and legends: * > 0.5 likely pathogenic; ** < 0.05 pathogenic; The synonym mutation (p.Arg437Arg) is interpreted by the SIFT only; VUS: variant of uncertain significance; HGMD: Human Gene Mutation Database; Ref No.: reference number in the text; ACMG: Americal College of Medical Genetics and Genomics
